# Prognostic significance of delirium subtypes in critically ill medical and surgical patients: a secondary analysis of a prospective multicenter study

**DOI:** 10.1186/s40560-022-00644-1

**Published:** 2022-12-20

**Authors:** Lisa Smit, Eveline J. A. Wiegers, Zoran Trogrlic, Wim J. R. Rietdijk, Diederik Gommers, Erwin Ista, Mathieu van der Jagt

**Affiliations:** 1 Department of Intensive Care Adults, Erasmus MC, University Medical Center, Doctor Molewaterplein 40, 3015 GD Rotterdam, The Netherlands; 2 Department of Public Health, Erasmus MC, University Medical Center, Rotterdam, The Netherlands; 3 Department of Hospital Pharmacy, Erasmus MC, University Medical Center, Rotterdam, The Netherlands; 4 Intensive Care Unit, Department of Pediatric Surgery, Erasmus MC-Sophia Children’s Hospital, University Medical Center, Rotterdam, The Netherlands; 5 Section of Nursing Science, Department of Internal Medicine, Erasmus MC, University Medical Center, Rotterdam, The Netherlands

**Keywords:** Delirium, Critical care, Intensive care unit, Mortality, Subtype, Critical care outcomes

## Abstract

**Background:**

The prognostic implication of delirium subtypes in critically ill medical and surgical patients is scarcely investigated. The objective was to determine how delirium subtypes are associated with hospital mortality and other clinical outcomes.

**Methods:**

We performed a secondary analysis on data from a prospective multicenter study aimed at implementation of delirium-oriented measures, conducted between 2012 and 2015 in The Netherlands. We included adults (≥ 18 years) admitted to the medical or surgical intensive care unit (ICU). Exclusion criteria were neurological admission diagnosis, persistent coma or ICU readmissions. Delirium was assessed using the Confusion Assessment Method-ICU or Intensive Care Delirium Screening Checklist, and delirium subtypes (hypoactive, hyperactive, or mixed) were classified using the Richmond Agitation–Sedation Scale. The main outcome was hospital mortality. Secondary outcomes were ICU mortality, ICU length of stay, coma, mechanical ventilation, and use of antipsychotics, sedatives, benzodiazepines and opioids.

**Results:**

Delirium occurred in 381 (24.4%) of 1564 patients (52.5% hypoactive, 39.1% mixed, 7.3% hyperactive). After case-mix adjustment, patients with mixed delirium had higher hospital mortality than non-delirious patients (OR 3.09, 95%CI 1.79–5.33, *p* = 0.001), whereas hypoactive patients did not (OR 1.34, 95%CI 0.71–2.55, *p* = 0.37). Similar results were found for ICU mortality. Compared to non-delirious patients, both subtypes had longer ICU stay, more coma, increased mechanical ventilation frequency and duration, and received more antipsychotics, sedatives, benzodiazepines and opioids. Except for coma and benzodiazepine use, the most unfavourable outcomes were observed in patients with mixed delirium.

**Conclusions:**

Patients with mixed delirium had the most unfavourable outcomes, including higher mortality, compared with no delirium. These differences argue for distinguishing delirium subtypes in clinical practice and future research.

*Trial registration* ClinicalTrials.gov NCT01952899.

**Supplementary Information:**

The online version contains supplementary material available at 10.1186/s40560-022-00644-1.

## Background

Delirium is a common form of vital organ failure in the intensive care unit (ICU), occurring in 30–70% of critically ill patients [1]. Its occurrence and duration are associated with longer hospital stay, higher healthcare costs, mortality, and long-term cognitive impairment [2–6]. Three distinct delirium subtypes have been described in the literature: hypoactive (or lethargic), hyperactive (or agitated), and mixed motor subtype [7, 8], indicating an alternating state between the other two subtypes. Delirium subtyping should, next to phenomenological differentiation, ideally have clear prognostic implications in order to direct prophylactic or therapeutic measures. A recent systematic review showed that it is still a matter of debate whether delirium subtypes are independently associated with mortality and ICU length of stay after correction for confounders, and that further studies are warranted [9]. Furthermore, only one study from this review reported outcomes in a mixed ICU [3].

Therefore, we aimed to study whether delirium subtypes were associated with adverse clinical outcomes through a secondary analysis on data from a large prospective multicenter implementation study focused on delirium-oriented measures in a mixed medical–surgical ICU population [10]. We hypothesized that patients with hypoactive delirium had highest mortality, followed by patients with mixed and hyperactive delirium [1].

## Methods

### Study design

We performed a secondary analysis on data collected of 5057 ICU patients for a prospective multicenter implementation study [ICU Delirium in Clinical Practice Implementation Evaluation (iDECePTIvE) study] [10]. The iDECePTIvE study was aimed to optimize adherence to delirium-oriented measures and was performed between 2012 and 2015 in six ICUs in the Rotterdam area, The Netherlands [10]. The implementation was constructed in three phases: after a baseline assessment (phase I), delirium assessment was implemented in all ICUs using either the Confusion Assessment Method for ICU (CAM-ICU) [11] or Intensive Care Delirium Screening Checklist (ICDSC), based on local preference [12]. Each participating ICU used one of these delirium assessment methods consistently and non-interchangeably. This baseline phase was followed by three further measurement periods (phases II–IV), focusing on the effects of implementation on adherence to daily assessments for delirium and to presence of other guideline recommendations from the Pain, Agitation and Delirium (PAD) guidelines issued by the Society of Critical Care Medicine [13]. Thus, phases II–IV implicated well embedded and structured three-times daily delirium assessments by ICU nurses.

After implementation of the PAD guideline recommendations [13], the iDECePTIvE study found improvements in delirium screening, the degree of physiotherapy and early mobilization, and the use of light sedation in ventilated patients [10]. The authors also reported a decrease in the use of benzodiazepines and delirium and coma duration.

The prospective data registry from the iDECePTIvE study was approved by the Medical Ethical Committee of the Erasmus University Medical Center (registration number: MEC-2012-063) and was not subjected to the Dutch law ‘Medical Research Involving Human Subjects’ (WMO). As such, the need for informed consent was waived. Involved investigators handled and analyzed anonymized data according to Dutch regulations. This study was reported using the Strengthening the Reporting of Observational Studies in Epidemiology (STROBE) statement [14].

### Study population

Inclusion criteria for this secondary analysis were adults (≥ 18 years) admitted to the medical or surgical ICU in phases II–IV, who had daily delirium assessments with either the CAM-ICU or ICDSC, and whose level of sedation was assessed daily with the Richmond Agitation–Sedation Scale (RASS) [15]. Patients with a primary neurological admission diagnosis had been excluded. Additional exclusion criteria were persisting coma (defined as RASS score − 4 or − 5 during the entire ICU stay) and ICU readmissions.

### Outcomes

The primary outcome was hospital mortality. Secondary outcomes were ICU mortality, ICU length of stay (in days), presence of coma during ICU stay (yes/no) and number of coma days, use of mechanical ventilation during ICU stay (yes/no) and number of ventilation days. Furthermore, we related the presence of delirium and the delirium subtypes to various management variables to gain further insight into their phenotypes: use of antipsychotics (yes/no) and number of days with haloperidol administration and haloperidol dose, and continuous intravenous administration during ICU stay of sedatives (yes/no), benzodiazepines (yes/no) and opioids (yes/no), and number of days of administration.

### Data collection

Patients were followed from ICU admission until hospital discharge. The following demographic data were prospectively collected in the Case Report Form or in the electronic patient data management system: age, sex, Acute Physiology and Chronic Health Evaluation (APACHE) IV score, ICU admission diagnosis (medical, elective surgery, or acute surgery), ICU length of stay, and ICU mortality and hospital mortality. Further, during ICU stay the following daily data were collected: mechanical ventilation, delirium assessments with CAM-ICU or ICDSC (three times daily), RASS scores, administration of antipsychotics [yes/no, including haloperidol (with dose), olanzapine, or quetiapine], and continuous intravenous administration during at least 2 h/day of sedatives (yes/no, including clonidine, dexmedetomidine or propofol), benzodiazepines (yes/no, including midazolam or lorazepam) and opioids (yes/no, including morphine, fentanyl or remifentanil).

### Definitions

We defined patients with ICU delirium as those with at least one positive delirium assessment with CAM-ICU or ICDSC during their ICU stay. We classified the delirium subtypes based on the combined results of the delirium assessment with either the CAM-ICU or ICDSC and the RASS score [15]. The RASS score ranges from − 5 (unarousable) to + 4 (combative), in which a RASS score of 0 indicates that the patient is calm and alert. If the RASS score was − 4 or − 5, delirium assessment was not possible [11]. Delirium was assessed with the CAM-ICU in three participating ICUs and in the fourth ICU the ICDSC was used. Delirium assessment was performed three times daily (once per 8-h shift) by ICU nurses, previously trained to use the CAM-ICU or ICDSC (depending on the hospital) [10, 16]. Hyperactive delirium was defined as a persistently positive RASS score (+ 1 to + 4) during all positive delirium assessments throughout the entire ICU stay, whereas a persistently negative or neutral RASS score (0 to − 3) at each positive delirium assessment was defined as hypoactive delirium [17]. Mixed delirium was defined as both hyper- and hypo-active delirium during ICU stay.

Further, regarding the secondary outcomes, the use of mechanical ventilation (yes/no) was defined as at least one mechanical ventilation day during ICU stay. Similarly, coma during ICU stay (yes/no) was defined as at least one coma day, with a coma day being a day on which patients had a RASS score of − 4 or − 5, but never obtained a RASS score of − 3 or higher, hindering delirium assessment. If patients had received antipsychotics (haloperidol, quetiapine, olanzapine), or continuous intravenous sedatives (clonidine, dexmedetomidine, propofol), benzodiazepines (midazolam or lorazepam) or opiates (morphine, fentanyl, remifentanil) for ≥ 2 h/day during ICU stay, they were defined accordingly (yes/no). Mean haloperidol daily doses were only reported for days on which patients received haloperidol.

### Statistical analyses

Continuous data were summarized as means with standard deviations or as medians with interquartile ranges (IQR), depending on distribution. Categorical variables were shown in frequencies and percentages. To assess differences between non-delirious patients and delirious patients, and between the delirium subtypes, *χ*^2^ tests were used for categorical data, independent *t*-tests for continuous normally distributed variables, and Mann–Whitney *U* tests for continuous non-normally distributed variables.

For the primary analysis, we used mixed-effects logistic regression models, with adjustment for prognostic factors related to delirium and mortality (APACHE IV, age, and ICU admission diagnosis) [18–20], and a random intercept for hospital. We tested for the interaction between delirium subtype and the APACHE IV score with the likelihood ratio test (LRT) and added the interaction term to the model when significant. Further, a propensity score model was used to match patients of the different subtypes with non-delirious patients. The propensity of having a specific delirium subtype was estimated using a mixed-effects multivariable logistic regression analysis with delirium subtype (yes/no) as an outcome, and the same independent variables as the primary logistic regression analysis. Patients with non-overlapping propensity scores (calliper of max 10%) were excluded for the effect analyses. Additionally, we performed a sensitivity analysis in order to investigate the interaction of delirium subtypes and concurrent guideline-based delirium-oriented measures, e.g., physiotherapy and early mobilization, and vice versa, by excluding patients who were included in phase II (i.e., the phase in which only delirium screening was implemented and not the guideline implementation measures including those related to physiotherapy and early mobilization).

For the secondary outcomes, logistic mixed-effects regression analyses were used to study ICU mortality, presence of coma, use of mechanical ventilation, antipsychotics, continuous intravenous sedatives, benzodiazepines and opioids, and linear mixed-effects regression analyses for ICU length of stay, number of delirium days, coma days and ventilation days, and the number of days on which antipsychotics (including haloperidol dose), sedatives, benzodiazepines or opioids were applied. We adjusted for the APACHE IV score, age, and ICU admission diagnosis, with a random effect for hospital. An interaction term of delirium subtype and APACHE IV score was added to the model when significant with the LRT.

A *p*-value < 0.05 was considered statistically significant. Descriptive analyses were performed using SPSS, version 24. Logistic and linear regression analyses and propensity score matching were performed in the R statistical software (version 4.1.0), for which multiple imputation was used to handle missing values, with use of the *mice* package in R [21].

## Results

**Fig. 1:**
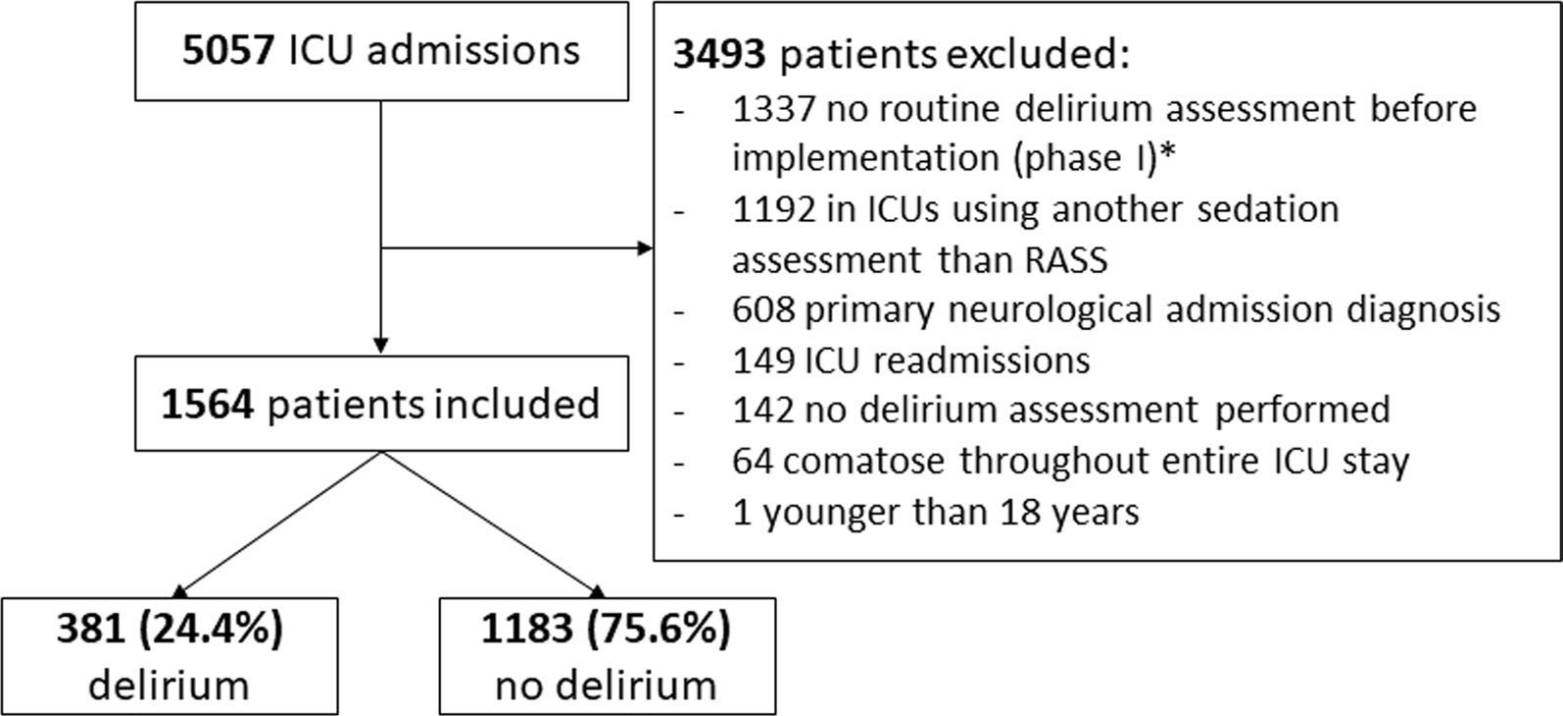
Flowchart of patient inclusion. *Based on the previous implementation study aimed at delirium-oriented measures [10]

The database of the implementation study included 4449 ICU patients. After applying exclusion criteria, 1564 patients were included, of whom 381 (24.4%) experienced delirium during ICU stay (Fig. 1). The median age was 65 years (IQR 52–74) and 952 (60.9%) patients were male.

### Demographic and clinical characteristics of delirious and non-delirious patients

Patients who developed delirium were older [67 years (IQR 56–75) versus 64 (IQR 51–74)], more often male (66.1% versus 59.2%), and more severely ill than non-delirious patients [APACHE IV score 72.5 (IQR 57–94.8) versus 51 (IQR 37–70)] (Table 1). Delirious patients were more likely to have been admitted with a medical diagnosis or for emergency surgery, whereas patients without delirium were more often admitted for elective surgery. In addition, delirious patients had a higher hospital mortality (21.4% versus 11.9%) and ICU mortality, and spent more days in the ICU than patients who did not develop delirium. Median delirium duration was 2 days (IQR 1–5). Delirious patients were more often comatose, had more coma days, were more often mechanically ventilated, including more time spent on mechanical ventilation, and were administered continuous intravenous sedatives (clonidine, dexmedetomidine, propofol), benzodiazepines, and opioids for ≥ 2 h/day more often and for a longer period.

**Table 1:** Demographic and clinical characteristics of included patients

Characteristic	No delirium *n* = 1183	Delirium *n* = 381	*p* value^a^
Age, years	64 (51–74)	67 (56–75)	0.001
Sex: male, *n* (%)	700 (59.2)	252 (66.1)	0.015
Admission diagnosis^b^			
Medical, *n* (%)	584 (51.4)	228 (65)	0.001
Elective surgery, *n* (%)	403 (35.5)	48 (13.7)	0.001
Emergency surgery, *n* (%)	149 (13.1)	75 (21.4)	0.001
APACHE IV score^c^	51 (37–70)	72.5 (57–94.8)	0.001
Hospital mortality, *n* (%)^b^	136 (11.9)	75 (21.4)	0.001
ICU mortality, *n* (%)^b^	94 (8)	51 (13.4)	0.001
ICU LOS, days	3 (2–4)	7 (4–14)	0.001
No. of delirium days	0 (0–0)	2 (1–4.5)	0.001
Coma, ever, *n* (%)^b^	157 (13.5)	137 (36.5)	0.001
No. of coma days^b,d^	1 (1–3)	2 (1–3)	0.020
Ventilation, ever, *n* (%)	518 (43.8)	300 (78.7)	0.001
Ventilation, days^d^	2 (1–3)	6 (3–12)	0.001
Continuous IV sedatives, ever, *n* (%)	415 (35.1)	281 (73.8)	0.001
Continuous IV sedatives, days^d^	2 (1–3)	5 (2–8)	0.001
Continuous IV benzodiazepines, ever, *n* (%)	118 (10)	112 (29.4)	0.001
Continuous IV benzodiazepines, days^d^	2 (1–3)	3 (2–5)	0.001
Continuous IV opioids, ever, *n* (%)	493 (41.7)	283 (74.3)	0.001
Continuous IV opioids, days^d^	2 (2–4)	5 (3–10)	0.001

Values are denoted as median (interquartile range) unless mentioned otherwise

*ICU* intensive care unit; *IV* intravenous; *LOS* length of stay

^a^Group differences were tested with Mann–Whitney *U* tests (continuous variables) or *χ*^2^ tests (categorical variables)

^b^Missing data were present for some patients: ICU admission diagnoses 77 (4.9%); APACHE IV score 132 (7.7%); hospital mortality 74 (4.7%); ICU mortality 1 (0.1%); coma, ever, and number of coma days 24 (1.5%)

^c^APACHE IV scores [20] range from 0 (best) to 286 (worst), based on the most abnormal values observed during 24 h following ICU admission

^d^Shown only for patients who had this characteristic ever during ICU stay

### Baseline characteristics of delirium subtypes

The hypoactive subtype occurred most frequently (*n* = 200; 52.5%), followed by the mixed (*n* = 149; 39.1%) and hyperactive subtype (*n* = 28; 7.3%). Delirium subtype was unknown in four patients (1%). The hyperactive group was excluded from the analysis, given its low incidence, precluding meaningful statistical analysis. As shown in Table 2, patients who experienced mixed delirium had highest disease severity, followed by the hypoactive and non-delirious patients. Patients with mixed delirium were older and more often male than non-delirious patients. Both subtypes were more likely to be admitted with a medical or emergency surgery diagnosis, but less likely for elective surgery.

**Table 2:** Baseline characteristics specified per delirium subtype

Characteristic	No delirium *n* = 1183	Delirium subtype^a^			
		Hypoactive *n* = 200	*p* value^b^	Mixed *n* = 149	*p* value^c^
Age, years	64 (51–74)	65 (56–74.8)	0.085	69 (57–75)	0.004
Sex: male, *n* (%)	700 (59.2)	125 (62.5)	0.38	102 (68.5)	0.029
Admission diagnosis^d^					
Medical, *n* (%)	584 (51.4)	116 (63.4)	0.003	92 (67.2)	0.001
Elective surgery, *n* (%)	403 (35.5)	30 (16.4)	0.001	15 (10.9)	0.001
Emergency surgery, *n* (%)	149 (13)	37 (20.2)	0.010	30 (21.9)	0.005
APACHE IV score^d,e^	51 (37–70)	70 (53–91.5)	0.001	79 (63–98)	0.001

Values are denoted as median (interquartile range) unless mentioned otherwise

^a^Hyperactive patients (*n* = 28) were excluded from the analysis, given its low incidence, precluding meaningful statistical analysis. For 4 patients delirium subtype was unknown

^b^Group differences between non-delirious patients and patients with the hypoactive subtype were tested with Mann–Whitney *U* tests (continuous variables) or *χ*^2^ tests (categorical variables)

^c^Group differences between non-delirious patients and patients with the mixed subtype were tested with Mann–Whitney *U* tests (continuous variables) or *χ*^2^ tests (categorical variables)

^d^Missing data were present for some patients: ICU admission diagnosis 76 (5.0%); APACHE IV score 129 (8.4%)

^e^APACHE IV scores [20] range from 0 (best) to 286 (worst), based on the most abnormal values observed during 24 h following ICU admission

### Association of delirium subtypes with hospital mortality

Patients with mixed-type delirium were more likely to die in hospital than patients without delirium [36/137 (26.3%) versus 136/1139 (11.9%); adjusted OR 3.09, 95%CI 1.79–5.33, *p* = 0.001], whereas patients with hypoactive delirium were not [31/183 (16.9%) versus 136/1139 (11.9%); adjusted OR 1.34, 95%CI 0.71–2.55, *p* = 0.37, Table 3]. Propensity score matching yielded comparable results (mixed vs. no delirium: OR 7.0, 95%CI 2.40–24.27, *p* = 0.001; hypoactive vs. no delirium: OR 1.64, 95%CI 0.65–4.36, *p* = 0.30, Additional file 1). The sensitivity analysis related to guideline implementation showed associations in the similar direction (Additional file 2).

**Table 3:** Association of delirium subtypes and covariates with hospital mortality

Variable	Mortality odds ratio (95% CI), unadjusted^a^	*p* value	Mortality odds ratio (95% CI), adjusted^b^	*p* value
Delirium subtypes (predictor of interest)^c^				
No delirium (reference)				
Hypoactive subtype	1.36 (0.91–2.05)	0.139	1.34 (0.71–2.55)	0.37
Mixed subtype	2.51 (1.66–3.78)	0.001	3.09 (1.79–5.33)	0.001
Covariates				
Age	1.04 (1.03–1.05)	0.001	1.03 (1.02–1.04)	0.001
APACHE IV^d^	1.05 (1.04–1.05)	0.001	1.05 (1.05–1.06)	0.001
Elective surgery (reference)				
Medical	5.84 (3.61–9.45)	0.001	2.55 (1.46–4.46)	0.001
Emergency surgery	4.06 (2.28–7.21)	0.001	1.91 (1.00–3.67)	0.05
No delirium*APACHE IV (reference)^d^				
Hypoactive subtype*APACHE IV	0.97 (0.95–0.99)	0.001	0.97 (0.96–0.99)	0.001
Mixed subtype*APACHE IV	0.96 (0.94–0.97)	0.001	0.96 (0.94–0.97)	0.001

^a^Analyzed with logistic regression analysis, after multiple imputation with the *mice* package in R

^b^Logistic mixed-effect model, adjusted for centered APACHE IV score and its interaction with delirium subtype, age and admission diagnosis, and a random intercept for hospital

^c^Hospital mortality in the no delirium group was 11.9% (*n* = 136), in patients with the hypoactive subtype 16.9% (*n* = 31) and in those with mixed subtype 26.3% (*n* = 36). Patients with hyperactive subtype (*n* = 28) were excluded from the analysis due to its low incidence, precluding meaningful statistical analysis. Hospital mortality occurred in 7 of these patients (25.9%). For 4 patients delirium subtype was unknown

^d^APACHE IV scores [20] range from 0 (best) to 286 (worst), based on the most abnormal values observed during 24 h following ICU admission. For the primary adjusted analysis, a centered APACHE IV score was used, as calculated by subtracting the mean APACHE IV score from the individual APACHE IV scores

In addition, we found that adding an interaction term between delirium subtype and APACHE IV score significantly improved the explanatory power of the model. The interaction term between APACHE IV and mixed-type (OR 0.96, 95%CI 0.94–0.97, *p* < 0.001) and hypoactive delirium (OR 0.97, 95%CI 0.96–0.99, *p* = 0.001) were both statistically significant. In Additional file 3, we provide the effect plot to help interpretation of the interaction term.

### Delirium subtypes and secondary outcomes

**Fig. 2:**
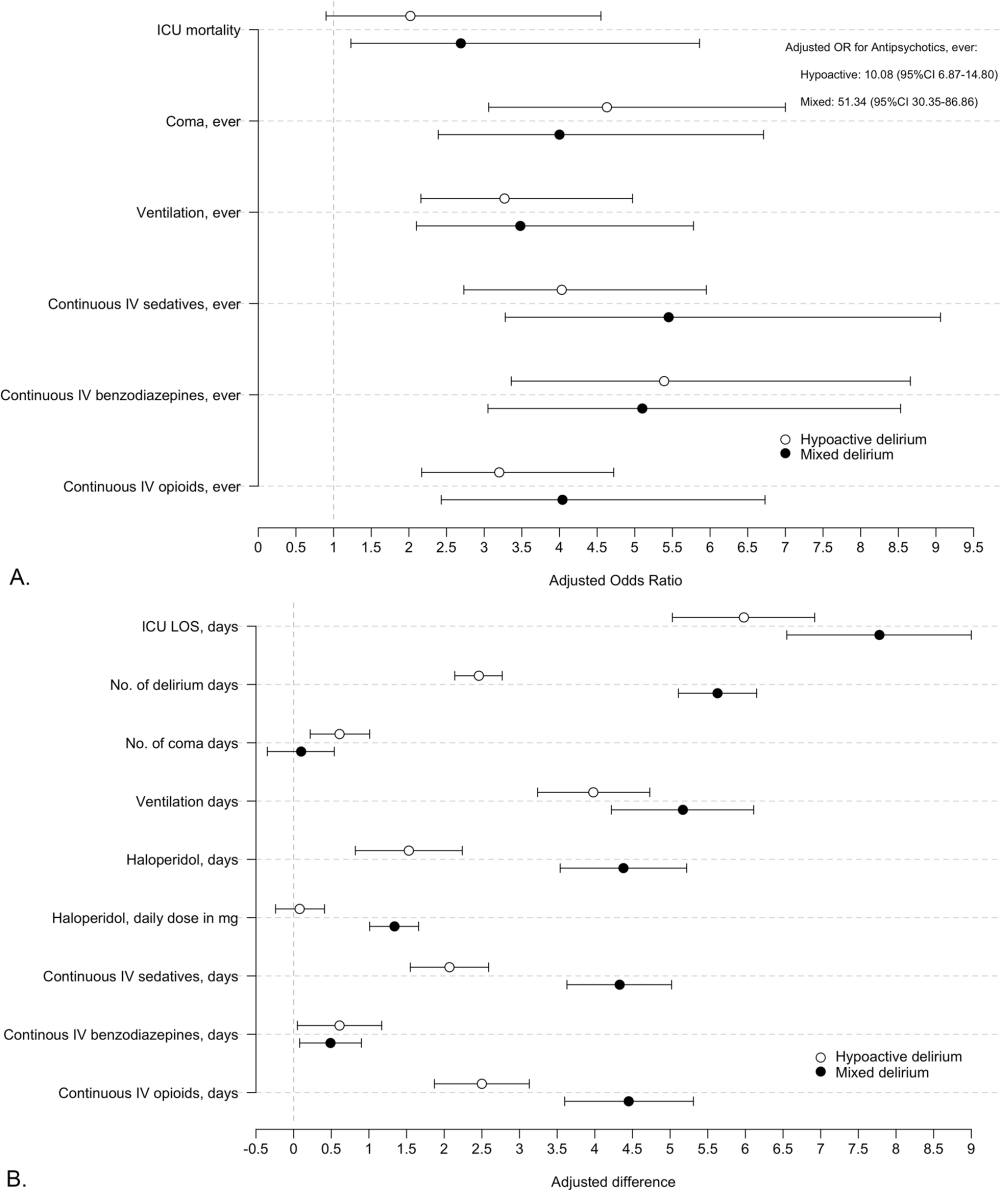
Association of delirium subtypes versus no delirium with secondary outcomes. *OR* odds ratio. **A** Shows the adjusted odds ratios with 95% confidence intervals for both delirium subtypes as compared to non-delirious patients, as analyzed with mixed-effect logistic regression models. These models were adjusted for age, admission diagnosis and APACHE IV score, and a random effect for hospital. An interaction term for delirium subtype with APACHE IV score, centered on its mean, was added to the model if significant. Since the odds ratio for outcome Antipsychotics, ever was larger than the other secondary categorical outcomes, this outcome was not shown graphically in the figure to improve visualization. The unadjusted and adjusted odds ratios are shown in Additional file 3. **B** Shows the adjusted differences with 95% confidence intervals of both delirium subtypes as compared to non-delirious patients, as analyzed with mixed-effect linear regression models. These models were adjusted for age, admission diagnosis and APACHE IV score, and a random effect for hospital. An interaction term for delirium subtype with APACHE IV score, centered on its mean, was added to the model if significant. The unadjusted and adjusted differences are shown in Additional file 3

When compared to patients without delirium, patients with mixed delirium had higher ICU mortality, but patients with hypoactive delirium did not (Fig. 2 and Additional file 4). Further, both the mixed and hypoactive subtypes were associated with longer ICU stay, more coma, and increased mechanical ventilation frequency and duration. Both subtypes had more and prolonged use of intravenous sedatives and opioids, and used intravenous benzodiazepines more frequently, with prolonged use in the mixed-delirium patients. Additionally, as compared to patients without delirium, patients of the mixed subtype had more delirium days and used more antipsychotics, including prolonged use and higher doses of haloperidol, than patients with hypoactive delirium. Except for coma and benzodiazepine use, the most unfavorable outcomes were observed in patients with mixed delirium.

## Discussion

In this secondary analysis of data from a large multicenter prospective cohort of medical and surgical ICU patients, we found that patients with mixed delirium had a substantial higher hospital mortality than patients with hypoactive delirium using non-delirious patients as the reference. After adjusting for relevant covariates and interaction terms and applying propensity matching, patients with mixed delirium generally had other more unfavorable short-term outcomes, among which ICU mortality and ICU length of stay, with varying treatment intensities between hypoactive and mixed delirium.

Our results regarding prevalence of delirium subtypes challenge findings from other large observational studies, in which mixed subtype was the most common [3, 17, 22, 23]. These studies predominantly involved elective surgery, neurology and neurosurgical patients [3, 22, 23], or patients younger than 65 years [17], while approximately half of our study population consisted of medical ICU patients being 65 years or older. As older patients in medical ICUs have a higher risk of developing hypoactive delirium [3, 24], this may explain why the incidence of this subtype was higher in our study than in previous studies.

A recent systematic review found that hypoactive delirium was associated with mortality in four studies [3, 7, 9, 25, 26]. We were not able to replicate these findings, probably because these studies were conducted in different countries, involved different ICU populations, used different delirium screening methods to classify delirium subtypes and were conducted before the PAD guideline recommendations were published [13]. Additionally, these studies reporting significant associations with mortality used univariate analyses and did not adjust for confounders. In contrast, we used mixed-effects logistic regression models with adjustment for prognostic factors related to delirium and mortality, and used validated delirium screening tools three-times daily (CAM-ICU and ICDSC) as recommended by the Pain, Agitation/Sedation, Delirium, Immobility, and Sleep Disruption (PADIS) guidelines of the Society of Critical Care Medicine [27]. Our finding that mixed delirium is associated with higher mortality risk is in accordance with another recent Dutch cohort study [22], conducted in mostly elective surgery patients. Therefore, our study adds to the current literature by using a multivariate analysis controlling for confounding and by focusing on mixed medical–surgical patients. This emphasizes the relevance for delirium subtyping in medical and surgical ICU patients given the prognostic significance. Nevertheless, the exact mechanisms responsible for increased mortality and other adverse outcomes, such as length of stay, in mixed delirium remain poorly understood, requiring further research, which in turn may help pinpoint more focused treatments.

Interestingly, we observed an interaction between delirium subtypes and disease severity as assessed with APACHE IV in explaining hospital mortality. Increasing disease severity was associated with highest slope in mortality rates in patients without delirium, followed by patients with hypoactive delirium and mixed delirium. Even though this interaction requires further research, a possible explanation may be that sedation administration in severely ill ICU patients may have hampered the detection of (hypoactive) delirium. As a result, delirium in severely ill patients may not have been recognized as such, or these patients did not develop delirium at all. In addition, mixed-delirium patients may definitely be noticed during their ICU stay as they express both hyper- and hypoactive symptoms. This may have led to more attention of the medical staff. In turn, this attention may have helped in more adequate or timely interventions in the treatment of these patients, and hence may have led to a decreasing influence of disease severity at ICU admission in explaining mortality risk. However, the present data do not allow for further causal inferences, and as such further investigation is needed.

Regarding other short-term clinical outcomes, previously published data on prognostic differences between delirium subtypes is conflicting [9]. Except for the presence and duration of coma and use of benzodiazepines, we found that patients with mixed subtype had the most unfavorable outcomes. Benzodiazepine administration is associated with increased delirium risk [19, 28], but less is known about the associated risk with certain delirium subtypes [29]. Sedation in general, and particularly benzodiazepines, may induce hypoactive symptoms or interfere with resolution of delirium. Hence, classifying rapidly reversible delirium as hypoactive delirium might have skewed our results towards better outcomes for hypoactive patients [22].

Further, we found that patients with mixed delirium were most likely to receive antipsychotics during ICU stay, with more days of haloperidol administration and higher doses administered. This reflects the recommendation of the recent PADIS guidelines of the Society of Critical Care Medicine [27] to use haloperidol only in case of distress, such as agitation and hyperactive symptoms. Nevertheless, this may have influenced our results related to the secondary outcomes, as haloperidol administration in delirious critically ill patients has been associated with more ventilations days and longer ICU stay [30].

Several other limitations should be considered. Mortality rates after hospital discharge are unknown in our study, hampering comparison to previously published longer-term mortality rates [2, 6, 7, 22]. Additionally, we were not able to determine if increased sedative and benzodiazepine use in the mixed and hypoactive subgroups were part of delirium treatment or if this actually caused more mixed or hypoactive delirium. For instance, use of sedatives in patients who initially experienced hyperactive delirium and who were sedated to a RASS score of − 2 were classified as mixed delirium. Further, even though delirium was assessed three times daily, there is a possibility that delirium was missed. Another limitation of the observational design is that, despite adjusting for multiple covariates, there may have been residual confounding.

Strengths of this study include the prospective multicenter study design, including medical and surgical patients, the sample size—which allowed correction for covariates—and the use of different statistical analyses. In addition, delirium screening was performed three times daily and the fact that our data reflect real-life clinical practice, rather than a strictly regulated setting as in clinical trials, may be regarded as a strength increasing external validity.

This study has several clinical implications. Delirium subtyping may be relevant to identify patients at increased risk of dying and other adverse health outcomes, although—given variable associations reported in the literature—these associations seem context specific. Still, subtyping delirium will be relevant in future studies aimed at assessing therapeutic interventions. Such research should also focus on longer-term outcomes, and on defining interactions of different ICU delirium subtypes with treatment effects.

## Conclusions

Critically ill patients with mixed but not hypoactive delirium had significantly worse hospital and ICU mortality than patients without delirium, and both had worse short-term outcomes, including longer ICU stay and mechanical ventilation in this study population. The mixed subtype appeared to have the worst prognosis, highlighting the need for delirium subtyping for delirium research and clinical management.

## Supplementary Information

**Additional file 1: Table S1.** Propensity score matching model: delirium subtypes versus no delirium and hospital mortality. **Table S2.** Characteristics of patients after propensity score matching: hypoactive patients versus non-delirious patients. **Table S3.** Characteristics of patients after propensity score matching: mixed patients versus non-delirious patients.

**Additional file 2.** Sensitivity analysis: association of delirium subtypes and covariates with hospital mortality in patients who received guideline implementation measures related to physiotherapy and early mobilization.

**Additional file 3: Figure S1.** Effect plot of delirium subtype and APACHE IV score.

**Additional file 4.** Association of delirium subtypes with secondary outcomes.

## Data Availability

All de-identified individual participant data that underlie the results reported in this article and the data dictionary will be shared with investigators with the purpose of individual participant data meta-analysis after approval from authors of this article and a signed data access agreement. All requests should be sent to m.vanderjagt@erasmusmc.nl.

## Abbreviations

APACHE: Acute Physiology and Chronic Health Evaluation
CAM-ICU: Confusion Assessment Method for ICU
CI: Confidence interval
ICDSC: Intensive Care Delirium Screening Checklist
ICU: Intensive care unit
iDECePTIvE: ICU Delirium in Clinical Practice Implementation Evaluation
OR: Odds ratio
PADIS: Pain, Agitation/Sedation, Delirium, Immobility, and Sleep Disruption
RASS: Richmond Agitation–Sedation Scale
STROBE: Strengthening the Reporting of Observational Studies in Epidemiology
