# Supplementary material for: Prognostic significance of delirium subtypes in critically ill medical and surgical patients: a secondary analysis of a prospective multicenter study
Source: J Intensive Care. 2022 Dec 20;10:54. doi: 10.1186/s40560-022-00644-1 (PMC9764534; doi:10.1186/s40560-022-00644-1)
Supplement: Supplementary file 1 — Additional file 1: Table S1. Propensity score matching model: delirium subtypes versus no delirium and hospital mortality. Table S2. Characteristics of patients after propensity score matching: hypoactive patients versus non-delirious patients. Table S3. Characteristics of patients after propensity score matching: mixed patients versus non-delirious patients. [file 40560_2022_644_MOESM1_ESM.docx]

**Additional files**

***Additional file 1: Table S1. Propensity score matching model: delirium subtypes versus no delirium and hospital mortality***

| **Delirium subtype** | **Odds Ratio (95%CI)** | **p value** |
| --- | --- | --- |
| Hypoactive subtype | 1.64 (0.65-4.36) | 0.300 |
| Mixed subtype | 7.00 (2.40-24.27) | 0.001 |

Reference was patients without delirium. Odds Ratios are the result of propensity score matched models for hypoactive vs no delirium and mixed vs no delirium, matched on age, gender, admission diagnosis and APACHE IV score (matching characteristics are shown in the other two tables in this Additional file). Both models included a significant interaction term for delirium subtype with APACHE IV score, centered on its mean.

***Additional file 1: Table S2. Characteristics of patients after propensity score matching: hypoactive patients versus non-delirious patients***

| **Characteristic ^a^** | **No delirium**  n = 224 | **Hypoactive**  n = 224 | ***p* value** |
| --- | --- | --- | --- |
| **Age, years** | 66.5 (55-75) | 65 (54.8-74) | 0.260 |
| **Gender: male, n (%)** | 123 (54.9) | 144 (64.3) | 0.054 |
| **Admission diagnosis** |  |  | 0.537 |
| Medical, n (%) | 128 (57.1) | 137 (61.2) |  |
| Elective surgery, n (%) | 37 (16.5) | 38 (17) |  |
| Emergency surgery, n (%) | 59 (26.3) | 49 (21.9) |  |
| **APACHE IV score ^b^** | 64 (48-86.3) | 68.5 (53.8-85.3) | 0.191 |

^a^ Values are denoted as median (interquartile range) unless mentioned otherwise

^b^ APACHE IV scores [20] range from 0 (best) to 286 (worst), based on the most abnormal values observed during 24 hours following ICU admission.

***Additional file 1: Table S3. Characteristics of patients after propensity score matching: mixed patients versus non-delirious patients***

| **Characteristic ^a^** | **No delirium**  n = 163 | **Mixed**  n = 163 | ***p* value** |
| --- | --- | --- | --- |
| **Age, years** | 68 (59-76.5) | 69 (58-75) | 0.778 |
| **Gender: male, n (%)** | 100 (61.3) | 111 (68.1) | 0.246 |
| **Admission diagnosis** |  |  | 0.068 |
| Medical, n (%) | 119 (73) | 108 (66.3) |  |
| Elective surgery, n (%) | 7 (4.3) | 18 (11) |  |
| Emergency surgery, n (%) | 37 (22.7) | 37 (22.7) |  |
| **APACHE IV score ^b^** | 75 (55-97) | 78 (63-98) | 0.298 |

^a^ Values are denoted as median (interquartile range) unless mentioned otherwise

^b^ APACHE IV scores [20] range from 0 (best) to 286 (worst), based on the most abnormal values observed during 24 hours following ICU admission.
