# Supplementary material for: Prognostic significance of delirium subtypes in critically ill medical and surgical patients: a secondary analysis of a prospective multicenter study
Source: J Intensive Care. 2022 Dec 20;10:54. doi: 10.1186/s40560-022-00644-1 (PMC9764534; doi:10.1186/s40560-022-00644-1)
Supplement: Supplementary file 2 — Additional file 2. Sensitivity analysis: association of delirium subtypes and covariates with hospital mortality in patients who received guideline implementation measures related to physiotherapy and early mobilization. [file 40560_2022_644_MOESM2_ESM.docx]

***Additional file 2. Sensitivity analysis: association of delirium subtypes and covariates with hospital mortality in patients who received guideline implementation measures related to physiotherapy and early mobilization.***

| **Variable** | **Mortality Odds Ratio (95% CI), unadjusted ^a^** | ***p* value** | **Mortality Odds Ratio (95% CI), adjusted ^b^** | ***p* value** |
| --- | --- | --- | --- | --- |
| *Delirium subtypes (predictor of interest)^c^* | | | | |
| No delirium (reference) |  | | | |
| Hypoactive subtype | 1.33 (0.76-2.35) | 0.324 | 1.26 (0.56-2.81) | 0.583 |
| Mixed subtype | 1.77 (0.95-3.30) | 0.076 | 1.90 (0.81-4.44) | 0.143 |
| *Covariates* | | | | |
| Age | 1.03 (1.02-1.04) | <.001 | 1. 02 (1.00-1.04) | 0.042 |
| APACHE IV ^d^ | 1.05 (1.04-1.05) | <.001 | 1.06 (1.05-1.07) | <.001 |
| Elective surgery (reference) |  | | | |
| Medical | 6.05 (3.16-11.58) | <.001 | 2.70 (1.29-5.64) | 0.009 |
| Emergency surgery | 3.20 (1.46-7.00) | 0.004 | 1. 82 (0.74-4.49) | 0.196 |
| No delirium * APACHE IV (reference)^d^ |  | | | |
| Hypoactive subtype * APACHE IV | 0.97 (0.95-0.99) | 0.011 | 0.97 (0.95-0.99) | 0.014 |
| Mixed subtype * APACHE IV | 0.96 (0.94-0.98) | <.001 | 0.96 (0.94-0.98) | 0.001 |

^a^ Analyzed with logistic regression analysis, after multiple imputation with the *mice* package in R.

^b^  Logistic mixed-effect model, adjusted for centered APACHE IV score and its interaction with delirium subtype, age and admission diagnosis, and a random intercept for hospital.

^c^ Hospital mortality in the no delirium group was 12.9% (n=78), in patients with the hypoactive subtype 16.7% (n=18) and in those with mixed subtype 20.3% (n=15).

^d^ APACHE IV scores ^20^ range from 0 (best) to 286 (worst), based on the most abnormal values observed during 24 hours following ICU admission. For the primary adjusted analysis, a centered APACHE IV score was used, as calculated by subtracting the mean APACHE IV score from the individual APACHE IV scores.
