# Supplementary figures and images for: Prognostic significance of delirium subtypes in critically ill medical and surgical patients: a secondary analysis of a prospective multicenter study
Source: J Intensive Care. 2022 Dec 20;10:54. doi: 10.1186/s40560-022-00644-1 (PMC9764534; doi:10.1186/s40560-022-00644-1)

***Additional file 3: Figure S1. Effect plot of delirium subtype and APACHE IV score
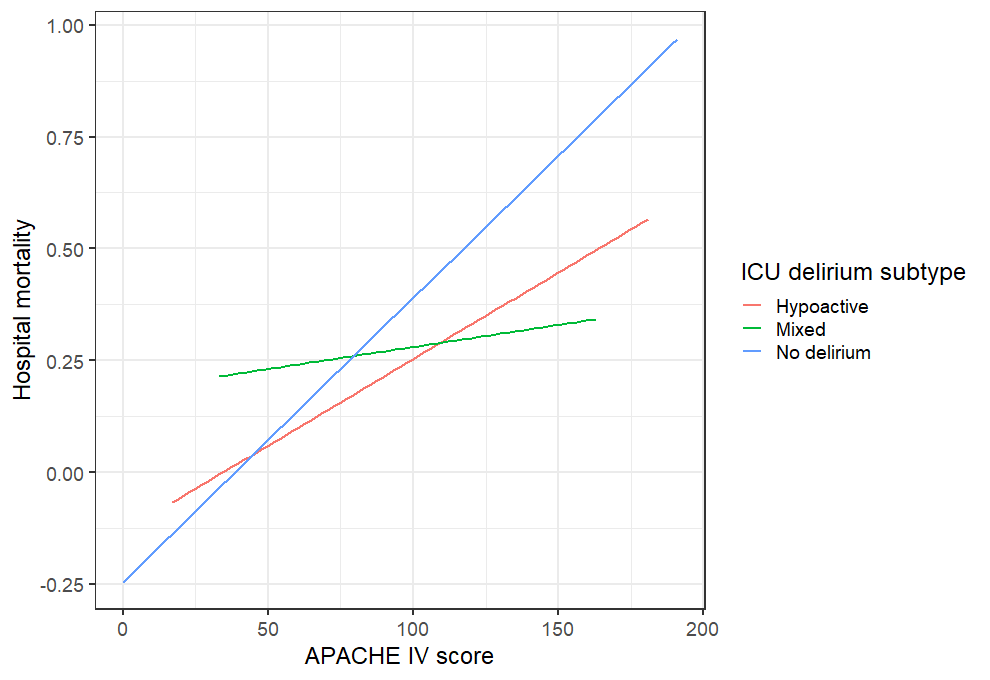
***

Supplement: Supplementary file 3 — Additional file 3: Figure S1. Effect plot of delirium subtype and APACHE IV score. [file 40560_2022_644_MOESM3_ESM.docx]
