# Supplementary material for: Prognostic significance of delirium subtypes in critically ill medical and surgical patients: a secondary analysis of a prospective multicenter study
Source: J Intensive Care. 2022 Dec 20;10:54. doi: 10.1186/s40560-022-00644-1 (PMC9764534; doi:10.1186/s40560-022-00644-1)
Supplement: Supplementary file 4 — Additional file 4. Association of delirium subtypes with secondary outcomes. [file 40560_2022_644_MOESM4_ESM.docx]

***Additional file 4. Association of delirium subtypes with secondary outcomes***

| **Outcome** | **Delirium subtype** | **Median (IQR) / n (%)** | **Unadjusted difference / Odds Ratio (95%CI) ^a^** | **p value** | **Adjusted difference / Odds Ratio (95%CI) ^a, b^** | **p value** |
| --- | --- | --- | --- | --- | --- | --- |
| **ICU mortality, n (%)** ^c^ | No delirium | 94 (8) | Reference |  | Reference |  |
|  | Hypoactive | 27 (13.5) | 1.81 (1.17-2.79) | 0.008 | 2.02 (0.90-4.55) | 0.093 |
|  | Mixed | 20 (13.4) | 1.66 (0.99-2.76) | 0.053 | 2.69 (1.23-5.86) | 0.013 |
|  | Hyperactive ^d^ | 3 (10.7) |  |  |  |  |
| **ICU LOS, days** | No delirium | 3 (2-4) | Reference |  | Reference |  |
|  | Hypoactive | 7 (4-13) | 7.13 (6.27-7.99) | 0.001 | 5.98 (5.03-6.92) | 0.001 |
|  | Mixed | 9 (5-16) | 9.29 (8.26-10.31) | <.001 | 7.78 (6.55-9.00) | 0.001 |
|  | Hyperactive ^d^ | 3.5 (2-5.8) |  |  |  |  |
| **No. of delirium days** | No delirium | 0 (0-0) | Reference |  | Reference |  |
|  | Hypoactive | 2 (1-3) | 2.82 (2.53-3.10) | 0.001 | 2.46 (2.14-2.77) | 0.001 |
|  | Mixed | 4 (2-7.5) | 5.78 (5.44-6.12) | 0.001 | 5.63 (5.11-6.15) | 0.001 |
|  | Hyperactive ^d^ | 1 (1-1) |  |  |  |  |
| **Coma, ever, n (%)** | No delirium | 157 (13.5) | Reference |  | Reference |  |
|  | Hypoactive | 76 (38.8) | 1.07 (2.94-5.63) | 0.001 | 4.63 (3.06-7.00) | 0.001 |
|  | Mixed | 52 (35.4) | 3.64 (2.49-5.33) | 0.001 | 4 (2.39-6.71) | 0.001 |
|  | Hyperactive ^d^ | 8 (28.6) |  |  |  |  |
| **No. of coma days** ^c, e^ | No delirium | 1 (1-3) | Reference |  | Reference |  |
|  | Hypoactive | 2 (1-3) | 0.71 (0.16-1.25) | 0.039 | 0.61 (0.22-1.01) ^f^ | 0.008 |
|  | Mixed | 2 (1-3) | 0.28 (-0.13-0.68) | 0.201 | 0.10 (-0.35-0.54) ^f^ | 0.681 |
|  | Hyperactive ^d^ | 1 (1-1) |  |  |  |  |
| **Ventilation, ever, n (%)** | No delirium | 518 (43.8) | Reference |  | Reference |  |
|  | Hypoactive | 159 (79.5) | 4.91 (3.44-7) | 0.001 | 3.27 (2.16-4.97) ^f^ | 0.001 |
|  | Mixed | 121 (81.2) | 5.32 (3.50-8.08) | 0.001 | 3.48 (2.10-5.78) ^f^ | 0.001 |
|  | Hyperactive ^d^ | 16 (57.1) |  |  |  |  |
| **Ventilation, days** ^e^ | No delirium | 2 (1-3) | Reference |  | Reference |  |
|  | Hypoactive | 5 (3-11) | 5.16 (4.46-5.85) | 0.001 | 3.98 (3.24-4.73) | 0.001 |
|  | Mixed | 8 (3-13.5) | 6.56 (5.71-7.40) | 0.001 | 5.17 (4.22-6.11) | 0.001 |
|  | Hyperactive ^d^ | 3 (2-7.5) |  |  |  |  |
| **Antipsychotics, ever, n (%)** | No delirium | 95 (8) | Reference |  | Reference |  |
|  | Hypoactive | 99 (49.5) | 10.80 (7.68-15.21) | 0.001 | 10.08 (6.87-14.80) ^f^ | 0.001 |
|  | Mixed | 124 (83.2) | 56.44 (34.85-91.41) | 0.001 | 51.34 (30.35-86.86) ^f^ | 0.001 |
|  | Hyperactive ^d^ | 14 (50) |  |  |  |  |
| **Haloperidol, days** ^e^ | No delirium | 2 (1-3) | Reference |  | Reference |  |
|  | Hypoactive | 4 (2-6.3) | 2.23 (1.56-2.89) | 0.001 | 1.53 (0.82-2.24) | 0.001 |
|  | Mixed | 5 (3-10) | 5.44 (4.75-6.12) | 0.001 | 4.38 (3.54-5.22) | 0.001 |
|  | Hyperactive ^d^ | 2 (1-4.5) |  |  |  |  |
| **Haloperidol, daily dose in mg** ^e^ | No delirium | 2.3 (1.5-3) | Reference |  | Reference |  |
|  | Hypoactive | 2.8 (2-3.1) | 0.16 (-0.21-0.53) | 0.425 | 0.08 (-0.24-0.41) | 0.630 |
|  | Mixed | 3.2 (2.7-4.8) | 1.34 (0.97-1.70) | 0.001 | 1.34 (1.01-1.66) | 0.001 |
|  | Hyperactive ^d^ | 2.5 (2-3) |  |  |  |  |
| **Continuous IV sedatives, ever, n (%)** | No delirium | 415 (35.1) | Reference |  | Reference |  |
|  | Hypoactive | 148 (74) | 5.12 (3.69-7.11) | 0.001 | 4.03 (2.73-5.95) ^f^ | 0.001 |
|  | Mixed | 117 (78.5) | 6.62 (4.37-10.04) | 0.001 | 5.45 (3.28-9.06) ^f^ | 0.001 |
|  | Hyperactive ^d^ | 14 (50) |  |  |  |  |
| **Continuous IV sedatives, days** ^e^ | No delirium | 2 (1-3) | Reference |  | Reference |  |
|  | Hypoactive | 4 (2-6.8) | 2.46 (1.98-2.93) | 0.001 | 2.07 (1.55-2.59) | 0.001 |
|  | Mixed | 6 (3-10.5) | 5.10 (4.55-5.66) | 0.001 | 4.33 (3.63-5.02) | 0.001 |
|  | Hyperactive ^d^ | 4 (2-6.8) |  |  |  |  |
| **Continuous IV benzodiazepines, ever, n (%)** | No delirium | 118 (10) | Reference |  | Reference |  |
|  | Hypoactive | 58 (29) | 3.68 (2.59-5.24) | 0.001 | 5.39 (3.36-8.66) ^f^ | 0.001 |
|  | Mixed | 47 (31.5) | 4.15 (2.80-6.14) | 0.001 | 5.10 (3.05-8.53) ^f^ | 0.001 |
|  | Hyperactive ^d^ | 6 (21.4) |  |  |  |  |
| **Continuous IV benzodiazepines, days** ^e^ | No delirium | 2 (1-3) | Reference |  | Reference |  |
|  | Hypoactive | 3 (2-5) | 0.55 (-0.06-1.15) | 0.128 | 0.61 (0.05-1.17) | 0.073 |
|  | Mixed | 3 (2-5) | 0.70 (0.22-1.19) | 0.019 | 0.49 (0.08-0.90) | 0.026 |
|  | Hyperactive ^d^ | 2 (1-5.5) |  |  |  |  |
| **Continuous IV opioids, ever, n (%)** | No delirium | 493 (41.7) | Reference |  | Reference |  |
|  | Hypoactive | 147 (73.5) | 3.97 (2.85-5.54) | 0.001 | 3.20 (2.17-4.72) ^f^ | 0.001 |
|  | Mixed | 117 (78.5) | 4.96 (3.29-7.49) | 0.001 | 4.04 (2.43-6.73) ^f^ | 0.001 |
|  | Hyperactive ^d^ | 17 (60.7) |  |  |  |  |
| **Continuous IV opioids, days** ^e^ | No delirium | 2 (2-4) | Reference |  | Reference |  |
|  | Hypoactive | 4 (3-9) | 3.15 (2.53-3.77) | 0.001 | 2.50 (1.87-3.13) | 0.001 |
|  | Mixed | 7 (3-11.5) | 5.04 (4.35-5.74) | 0.001 | 4.45 (3.60-5.31) | 0.001 |
|  | Hyperactive ^d^ | 3 (1.5-6.0) |  |  |  |  |

^a^ Denoted as differences for continuous variables and as odds ratios for categorical variables.

^b^ Analyzed with mixed-effects logistic or linear regression analyses, adjusted for age, APACHE IV score, admission category and an interaction term for delirium subtype and APACHE IV score (unless mentioned otherwise), with a random effect for hospital.

^c^ Missing data were present for some patients: ICU mortality 1 (<0.1%), number of coma days 24 (1.4%).

^d^ Excluded from the analysis, given its low incidence, precluding meaningful statistical analysis

^e^ Shown only for patients who had this characteristic ever during ICU stay.

^f^ Interaction term for delirium subtype * APACHE IV score was not significant, and hence was not added to the adjusted model related to this secondary outcome.
